# Comparative analysis of prophages in *Streptococcus mutans* genomes

**DOI:** 10.7717/peerj.4057

**Published:** 2017-11-17

**Authors:** Tiwei Fu, Xiangyu Fan, Quanxin Long, Wanyan Deng, Jinlin Song, Enyi Huang

**Affiliations:** 1 College of Stomatology, Chongqing Medical University, Chongqing Key Laboratory for Oral Diseases and Biomedical Sciences, Chongqing Municipal Key Laboratory of Oral Biomedical Engineering of Higher Education, Chongqing, China; 2 School of Biological Science and Technology, University of Jinan, Jinan, China; 3 Key Laboratory of Molecular Biology for Infectious Diseases of Ministry of Education, Chongqing Medical University, Chongqing, China; 4 Key Laboratory of Molecular Biology for Infectious Diseases (Ministry of Education), Institute for Viral Hepatitis, Department of Infectious Diseases, The Second Affiliated Hospital, Chongqing Medical University, Chongqing, China

**Keywords:** Comparative genomics, *Streptococcus mutans*, Prophages

## Abstract

Prophages have been considered genetic units that have an intimate association with novel phenotypic properties of bacterial hosts, such as pathogenicity and genomic variation. Little is known about the genetic information of prophages in the genome of *Streptococcus mutans*, a major pathogen of human dental caries. In this study, we identified 35 prophage-like elements in *S. mutans* genomes and performed a comparative genomic analysis. Comparative genomic and phylogenetic analyses of prophage sequences revealed that the prophages could be classified into three main large clusters: Cluster A, Cluster B, and Cluster C. The *S. mutans* prophages in each cluster were compared. The genomic sequences of phismuN66-1, phismuNLML9-1, and phismu24-1 all shared similarities with the previously reported *S. mutans* phages M102, M102AD, and ϕAPCM01. The genomes were organized into seven major gene clusters according to the putative functions of the predicted open reading frames: packaging and structural modules, integrase, host lysis modules, DNA replication/recombination modules, transcriptional regulatory modules, other protein modules, and hypothetical protein modules. Moreover, an integrase gene was only identified in phismuNLML9-1 prophages.

## Introduction

A prophage is a temperate bacteriophage genome integrated into a host bacterial DNA chromosome, which has the ability to enter a lysogenic state and replicate vertically with the host (St-Pierre & Endy, 2008). Prophages are an important source of virulence factors and other determinants that affect bacterial pathogenesis. Whole genome sequencing projects and comparative genomic analysis have revealed that prophage sequences are widespread among bacterial genomes, such as *Moraxella catarrhalis* (Ariff et al., 2015), *Enterococcus spp.* (Duerkop, Palmer & Horsburgh, 2014), *Lactococcus spp.* (Ventura et al., 2007), *Mycobacterium spp.* (Fan et al., 2014), and *Streptococcus suis* (Tang et al., 2013). Yet very little is known about *Streptococcus mutans* prophages.

Dental caries are the most prevalent dental disease and an important public health problem worldwide (Kidd & Fejerskov, 2013). The development of carious lesions stems from a dynamic process mediated by acid produced by cariogenic bacteria, such as *Streptococcus mutans*, *Streptococcus sobrinus*, and *Lactobacilli*, eventually resulting in de-mineralization and damage to the tooth structure (Argimon et al., 2014; Ericson et al., 2003). *S. mutans* are Gram-positive and biofilm-forming bacteria that can adhere to the tooth surface and contribute to dental plaque. *S. mutans* is the major pathogen responsible for dental caries in humans (Freires et al., 2017; Motegi et al., 2006). To the best of our knowledge, there have not been any reports describing *S. mutans* prophages, and only five *S. mutans* phages have been isolated. Three of them, M102, M102AD, and ϕAPCM01, have been sequenced (Dalmasso et al., 2015; Delisle et al., 2012; Van der Ploeg, 2007). Two other *S. mutans* phages, f1 and e10, have previously been isolated and tested for their host range and morphology, but not sequenced (Delisle & Rostkowski, 1993). Currently, there are 171 *S. mutans* genomic sequences in the National Center for Biotechnology Information (NCBI) database. Genomic sequencing of *S. mutans* has made it possible to identify prophages and perform comparative genomic analysis of prophage sequences and organization.

In this study, we screened all available complete *S. mutans* genomic sequences and identified 35 prophage-like elements present in these sequences. We also report the functional features of the intact prophages in comparison with another *S. mutans* phage, M102AD. Comparative genomic analysis and genome content analysis of *S. mutans* prophages were performed, and genetic information was analyzed.

## Materials and Methods

### Data collection and prophage sequence analyses

In total, 171 *S. mutans* genomes were obtained from NCBI. For prophage identification, tools such as PhiSpy (Akhter, Aziz & Edwards, 2012) and VirSorter (Roux et al., 2015) have been published as fast, relatively straight forward, and easier to use. We detected putative prophage DNA sequence data using the previously reported PHAge Search Tool Enhanced Release (PHASTER) method. PHASTER (http://phaster.ca/) was used to analyze bacterial genomes to identify and annotate putative prophage sequences (Arndt et al., 2016; Fan et al., 2016).

### Genomic and comparative genomic analyses of *S. mutans* prophages

Dot plot comparisons of *S. mutans* prophage genomes were performed using Geneious v.10.0.5 (http://www.geneious.com, Kearse et al., 2012). Prophage open reading frames (ORFs) were predicted using PHASTER, GeneMarkS, and BLASP (Zhu, Lomsadze & Borodovsky, 2010). The genomic organization of *S. mutans* prophage genome maps was constructed by SnapGene or SnapGene Viewer (http://www.snapgene.com; GSL Biotech, Chicago, IL, USA) (Sturmberger et al., 2016). The genomes comparison was performed on the DNA level with BLASTn (http://blast.ncbi.nlm.nih.gov/Blast.cgi) based on the percentage of sequence identity, and results were analyzed using Artemis Comparison Tool software (Carver et al., 2008). Default settings were used in all software.

### Phylogenetic analysis

Alignments of *S. mutans* phage and prophage genomic sequences were performed using MEGA version 7.0 (Tamura et al., 2007). Phylogenetic analysis was performed by the neighbor-joining (NJ) method and visualized using MEGA software. Phylogenetic distances were calculated by the NJ method using the same software.

## Results and Discussion

### Prophages are prevalent in *S. mutans* genomes

Data from 171 available whole *S. mutans* genomic sequences were downloaded from the NCBI website and analyzed (Table S1). The PHASTER web server was used to identify and annotate putative prophage regions within all *S. mutans* genomes. Thirty-five prophage-like elements were identified from 24 *S. mutans* genomes (13.45%) (Table 1). The genome sizes of *S. mutans* prophages ranged from approximately 4.7 to 68.2 kilobases, and the GC content varied between 35.62 and 44.56%. Only three prophages (phismuNLML9-1, phismuN66-1, and phismu24-1) appeared to represent complete phages with intact genomes. The remaining prophages were incomplete or questionable. The genomes of *S. mutans* NG8, *S. mutans* R221, *S. mutans* M230, *S. mutans* N29, *S. mutans* NLML9, *S. mutans* N66, and *S. mutans* 24 were polylysogenic. As many *S. mutans* genomes have prophages, and clustered regularly interspaced palindromic repeats (CRISPR)/CRISPR-associated (Cas9) can be viewed as a prokaryotic immune system that confers resistance to foreign genetic elements such as phages (Barrangou et al., 2007), we predict that CRISPR may be present in *S. mutans* genomes.

**Table 1:** Summary of genomic features of prophages in *S. mutans* genomes.

**Number**	**Prophages**	**Host**	**Accession/GI number**	**Size**	**No. CDS**	**Completeness**	**Region position**	**G + C percentage**	**References**
1	phismuU159	*Streptococcus mutans* UA159	NC_004350.2	11.6 Kb	17	Incomplete	1777567–1789234	35.62%	This study
2	phismu UA159-FR	*Streptococcus mutans* UA159-FR	NZ_CP007016.1	11.6 Kb	12	Incomplete	1776275–1787942	35.62%	This study
3	phismu NG8-1	*Streptococcus mutans* NG8	NZ_CP013237.1	8.3 Kb	6	Incomplete	178504–186829	37.77%	This study
4	phismu NG8-2	*Streptococcus mutans* NG8	NZ_CP013237.1	9.4 Kb	9	Incomplete	590838–600315	37.55%	This study
5	phismu NG8-3	*Streptococcus mutans* NG8	NZ_CP013237.1	6.8 Kb	7	Incomplete	737516–744365	38.31%	This study
6	phismu NG8-4	*Streptococcus mutans* NG8	NZ_CP013237.1	9.3 Kb	9	Incomplete	1186265–1195569	37.39%	This study
7	phismu NG8-5	*Streptococcus mutans* NG8	NZ_CP013237.1	10.2 Kb	9	Incomplete	1217598–1227802	38.30%	This study
8	phismu NG8-6	*Streptococcus mutans* NG8	NZ_CP013237.1	8.8 Kb	7	Incomplete	1551469–1560279	41.32%	This study
9	phismu NG8-7	*Streptococcus mutans NG8*	NZ_CP013237.1	11.5 Kb	12	Incomplete	1601756–1613341	33.74%	This study
10	phismu R221-1	*Streptococcus mutans* R221	AHRG00000000.1	8.6 Kb	12	Incomplete	1956467–1965159	37.36%	This study
11	phismu R221-2	*Streptococcus mutans* R221	AHRG00000000.1	4.7 Kb	12	Incomplete	1977201–1981928	38.11%	This study
12	phismu M230-1	*Streptococcus mutans* M230	AHRH00000000.1	25.5 Kb	24	Incomplete	1785712–1811298	37.88%	This study
13	phismu M230-2	*Streptococcus mutans* M230	AHRH00000000.1	7.2 Kb	18	Incomplete	1901561–1908828	40.24%	This study
14	phismu N29-1	*Streptococcus mutans* N29	AHRY00000000.1	29.7 Kb	20	Incomplete	1128124–1157831	36.66%	This study
15	phismu N29-2	*Streptococcus mutans* N29	AHRY00000000.1	26.7 Kb	7	Incomplete	1931418–1958213	37.56%	This study
16	phismu NFSM1	*Streptococcus mutans* NFSM1	AHSG00000000.1	10.3 Kb	12	Incomplete	1966153–1976539	38.43%	This study
17	phismu SF14	*Streptococcus mutans* SF14	AHSQ00000000.1	19.5 Kb	8	Incomplete	1677151–1696732	34.28%	This study
18	phismu U2A	*Streptococcus mutans* U2A	AHSU00000000.1	4.7 Kb	12	Questionable	2094437–2099191	42.29%	This study
19	phismu21	*Streptococcus mutans* 21	AHSZ00000000.1	26.3 Kb	21	Incomplete	1953048–1979373	36.14%	This study
20	phismu B	*Streptococcus mutans* B	AHTB00000000.1	21.2 Kb	25	Incomplete	1632476–1653683	35.81%	This study
21	phismu SM1	*Streptococcus mutans* SM1	AHTD00000000.1	21 Kb	11	Incomplete	1627422–1648498	38.20%	This study
22	phismu 3SN1	*Streptococcus mutans* 3SN1	AHRM00000000.1	18.1 Kb	22	Incomplete	1972568–1990744	38.54%	This study
23	phismu11SSST2	*Streptococcus mutans* 11SSST2	AHRP00000000.1	6.9 Kb	12	Incomplete	1947597–1954585	35.91%	This study
24	phismu NLML9-1	*Streptococcus mutans* NLML9	AHSJ00000000.1	68.2 Kb	61	Intact	56412–124703	35.60%	This study
25	phismu NLML9-2	*Streptococcus mutans* NLML9	AHSJ00000000.1	5.6 Kb	9	Incomplete	1953105–1958800	44.56%	This study
26	phismu N66-1	*Streptococcus mutans* N66	AHSM00000000.1	28.2 Kb	29	Intact	1120823–1149060	35.12%	This study
27	phismu N66-2	*Streptococcus mutans* N66	AHSM00000000.1	6.4 Kb	10	Incomplete	1907902–1914358	35.74%	This study
28	phismu W6	*Streptococcus mutans* W6	AHSO00000000.1	10.2 Kb	11	Incomplete	1738562–1748818	35.53%	This study
29	phismu SF1	*Streptococcus mutans* SF1	AHSP00000000.1	20.8 Kb	23	Incomplete	1543665–1564492	38.02%	This study
30	phismu ST6	*Streptococcus mutans* ST6	AHST00000000.1	16.4 Kb	25	Questionable	1863533–1879945	37.99%	This study
31	phismu NLML1	*Streptococcus mutans* NLML1	AHSW00000000.1	19.9 Kb	11	Incomplete	2015472–2035376	36.58%	This study
32	phismu 24-1	*Streptococcus mutans* 24	AHTE00000000.1	31.2 Kb	38	Intact	1547238–1578459	35.98%	This study
33	Phismu 24-2	*Streptococcus mutans* 24	AHTE00000000.1	22.3 Kb	14	Questionable	1982123–2004427	37.03%	This study
34	Phismu 5DC8	*Streptococcus mutans* 5DC8	AOBX00000000.1	11.6 Kb	16	Incomplete	1741331–1753010	35.62%	This study
35	phismu KK21	*Streptococcus mutans* KK21	AOBY00000000.1	11.6 Kb	16	Incomplete	1781240–1792907	35.62%	This study

### Sequence similarities among *S. mutans* prophages

**Figure 1:**
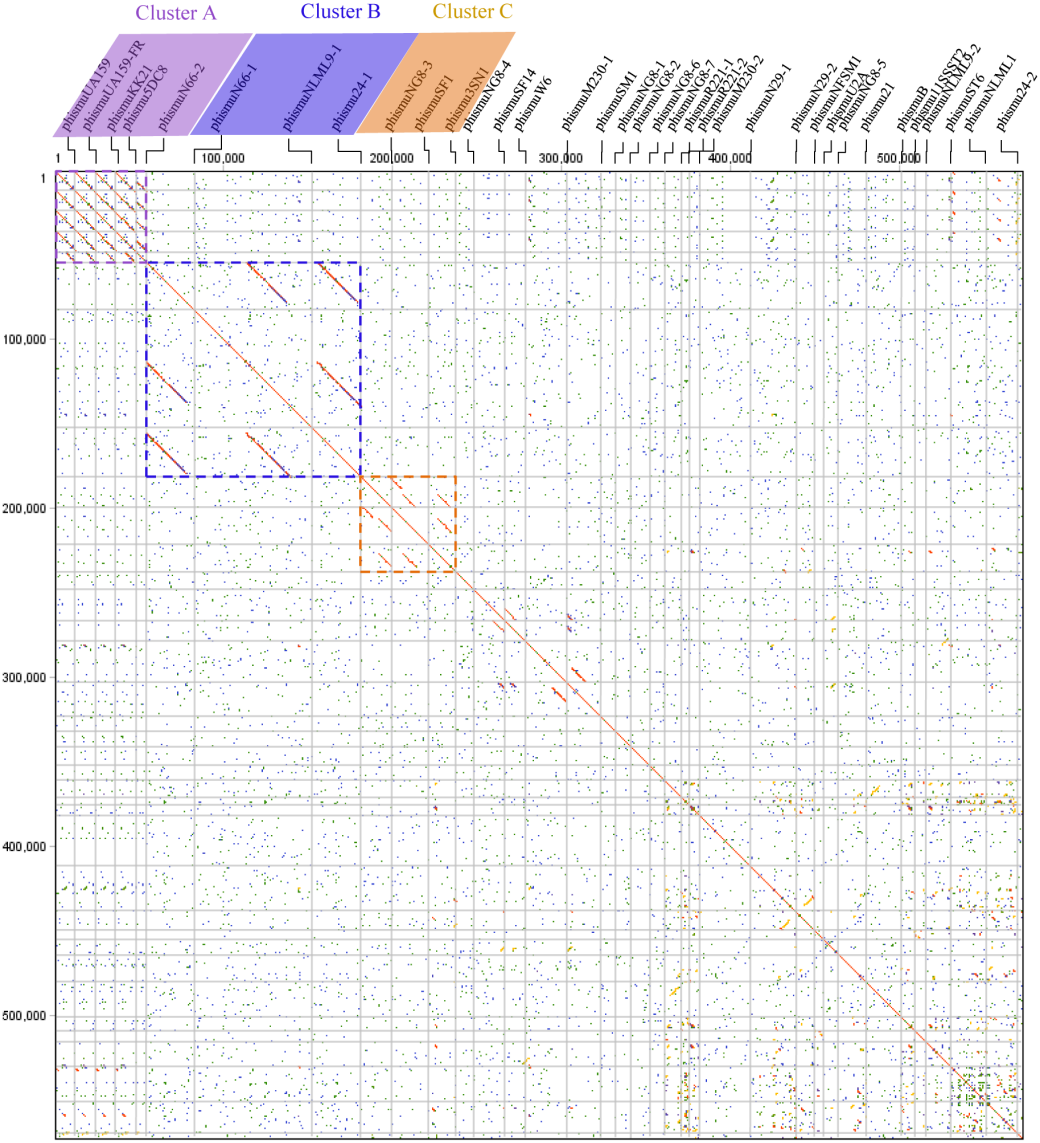
Dot plot matrix comparison calculated for the genomes of 35 Streptococcus mutans prophages. Prophage genome comparison of full genomes; the main diagonal represents the alignment of a sequence with itself. Regions of local similarity or repetitive sequences give rise to further diagonal matches in addition to the central diagonal, which indicates high similarity of the prophages. The *x*- and *y*-axis indicate full genomic sequence comparisons of prophage genomes. The length of the lines represents the length of the prophage genomes. The dot plot matrix was calculated using Geneious (http://www.geneious.com, Kearse et al., 2012).

Comparative genomic analyses were carried out through dot plots of 35 *S. mutans* prophage genomes. Dot plot analysis revealed that *S. mutans* prophages sorted into three clusters based on genomic similarities: Cluster A, Cluster B, and Cluster C (Fig. 1). Cluster A contains phismuU159, phismuUA159-FR, phismuKK21, phismu5DC8, and phismuN66-2. Cluster B contains phismuN66-1, phismuNLML9-1, and phismu24-1. Cluster C contains phismuNG8-3, phismuSF1, and phismu3SN1. Other *S. mutans* prophages, such as phismuSF14 and phismuW6, phismuM230-1 and phismuSM1, phismuN29-1 and phismuNFSM1, and phismu21 and phismuB, shared small fragments of genomic similarity, but could not be grouped into a cluster.

**Figure 2:**
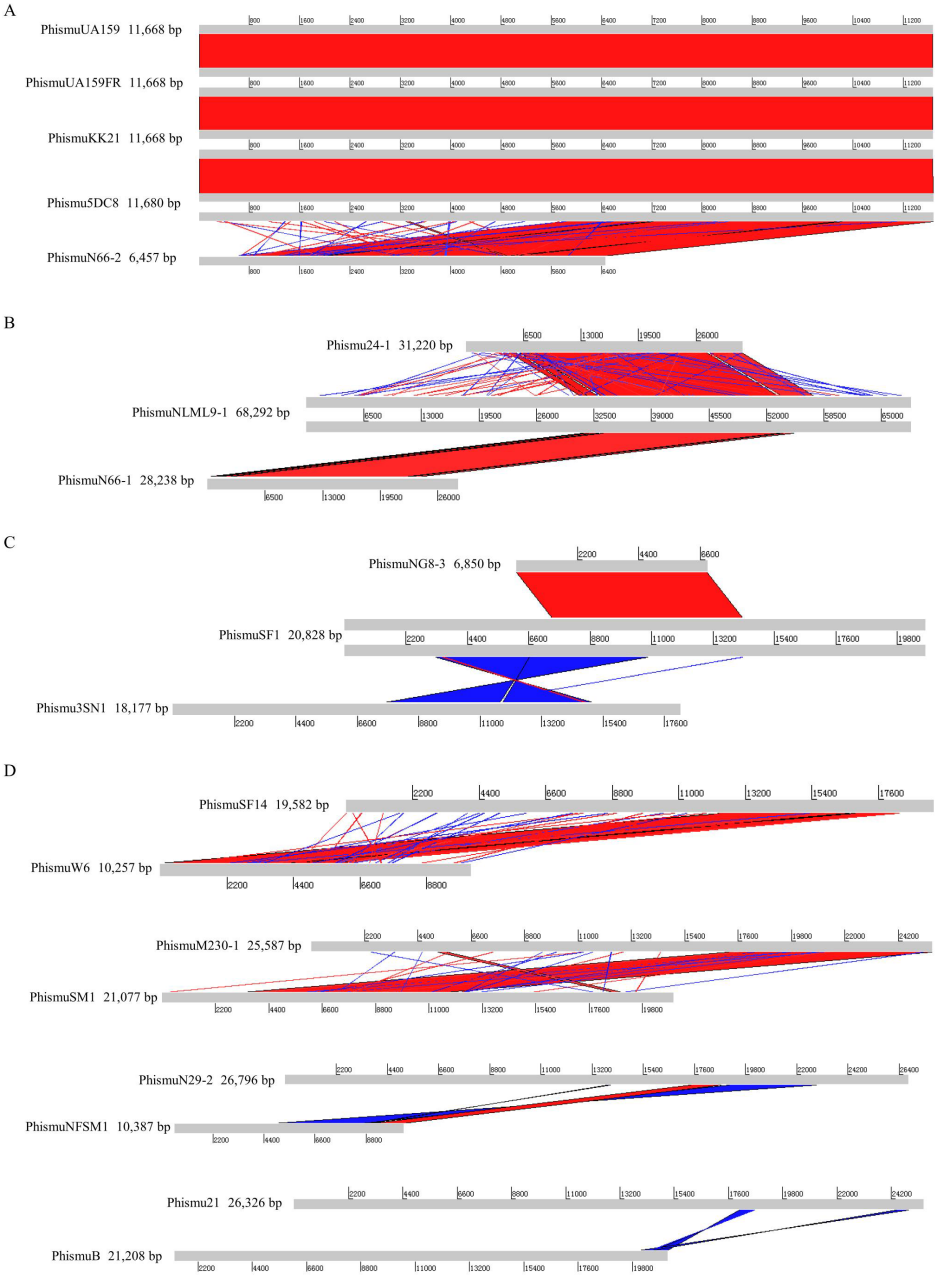
Genomic sequence comparison of S. mutans prophages. Comparisons were performed using the BLASTn and Artemis Comparison Tool visualization programs. Forward and reverse matches are colored red and blue, respectively.

### Comparative analysis of *S. mutans* prophages

Based on the similarities of their genomes, *S. mutans* prophages were divided into three clusters. Cluster A prophages (phismuUA159, phismuUA159-FR, phismuKK21, and phismu5DC8) shared 100% identity with one another at the genomic sequence level. Prophage phismuN66-2 shared one major region (5,854 base pairs [bp]) of sequence similarity, with 99.98% identity (Fig. 2A). Cluster B prophages (phismuN66-1, phismuNLML9-1, and phismu24-1) possessed at least 83% identity with one another, as determined by multiple genomic sequence alignments (Fig. 2B). In addition, BLASTn comparison of Cluster C (phismuSF1, phismuNG8-3, and phismu3SN1) revealed one segment (6,852 bp) with identity greater than 99% between phismuSF1 and phismuNG8-3 (Fig. 2C). Sequence comparison showed that phismuSF1 and phismu3SN1 shared two major regions of reverse complementary sequences (4,253 and 3,352 bp) with a similarity of 99.41 and 98.6% identity, respectively (Fig. 2C). Prophages belonging to individual clusters were more closely related to one another than to phages in the other clusters (Fig. S1). Other *S. mutans* prophages such as phismuSF14 and phismuW6 shared two major sequence (4,199 and 1,427 bp) similarities of 99.19 and 98.8% identity, respectively. PhismuM230-1 and phismuSM1 shared one major sequence (8,367 bp) similarity of 99.19% identity. PhismuN29-1 and phismuNFSM1 shared one major reverse complementary sequence (3,765 bp) similarity of 99.15% identity and one small sequence (1,291 bp) similarity of 99.15% identity. Phismu21 and phismuB shared two small reverse complementary sequence (566 and 471 bp) similarities of 99.82 and 97.66% identity, respectively (Fig. 2D).

### Phylogeny of *S. mutans* prophages

**Figure 3:**
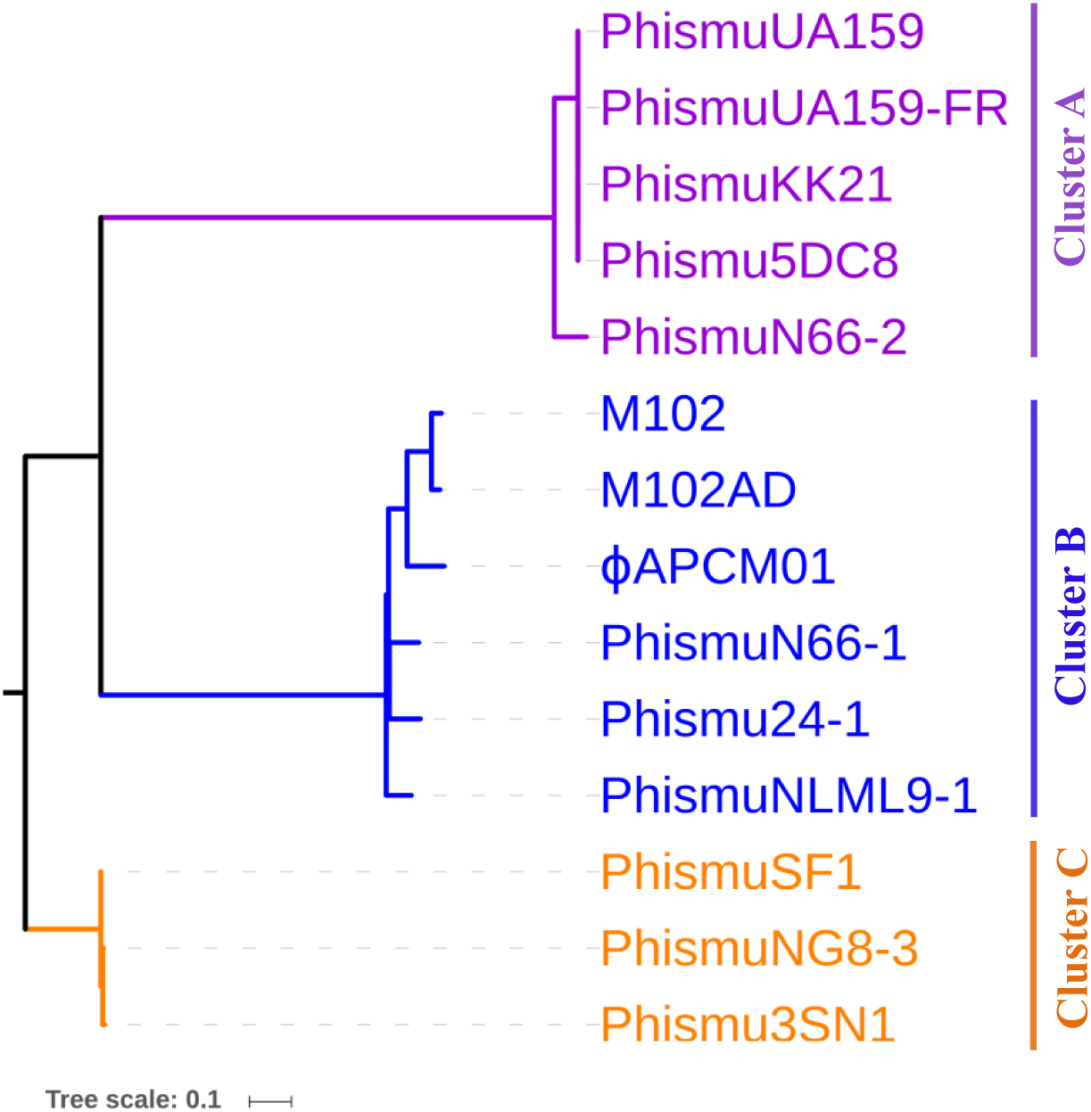
Phylogenetic analysis of S. mutans (pro)phage genomic sequence alignment. A phylogenetic tree of the genomic sequences of *S. mutans* (pro)phages was constructed using the neighbor-joining method. The phages were classified into three clusters, showing the phylogenetic relationships among the (pro)phage genomic sequences. Scale bar indicates 0.1 substitutions per site.

To understand how *S. mutans* prophages are related to one another, a genome phylogenetic tree was constructed based on the complete genomic sequences of some *S. mutans* prophages, including Cluster A, Cluster B, Cluster C, and three previously reported *S. mutans* phages M102 (Delisle & Rostkowski, 1993), M102AD (Delisle et al., 2012), and ϕAPCM01 (Dalmasso et al., 2015). The same grouping patterns and relationships were observed among the three large clusters (Clusters A, B, and C) as in the phylogenetic tree (Fig. 3). Cluster A consisted of phismuUA159, phismuUA159-FR, phismuKK21, phismu5DC8, and phismuN66-2. PhismuUA159, phismuUA159-FR, phismuKK21, and phismu5DC8 showed the closest distinct branch in the phylogenetic tree. Cluster B consisted of M102, M102AD, ϕAPCM01, phismuN66-1, phismuNLML9-1, and phismu24-1. These results suggested that the three sequenced *S. mutans* phages (M102, M102AD, and ϕAPCM01) belonged to Cluster B. Cluster C consisted of phismuSF1, phismuNG8-3, and phismu3SN1.

### Comparative analysis between M102AD and *S. mutans* prophages

**Figure 4:**
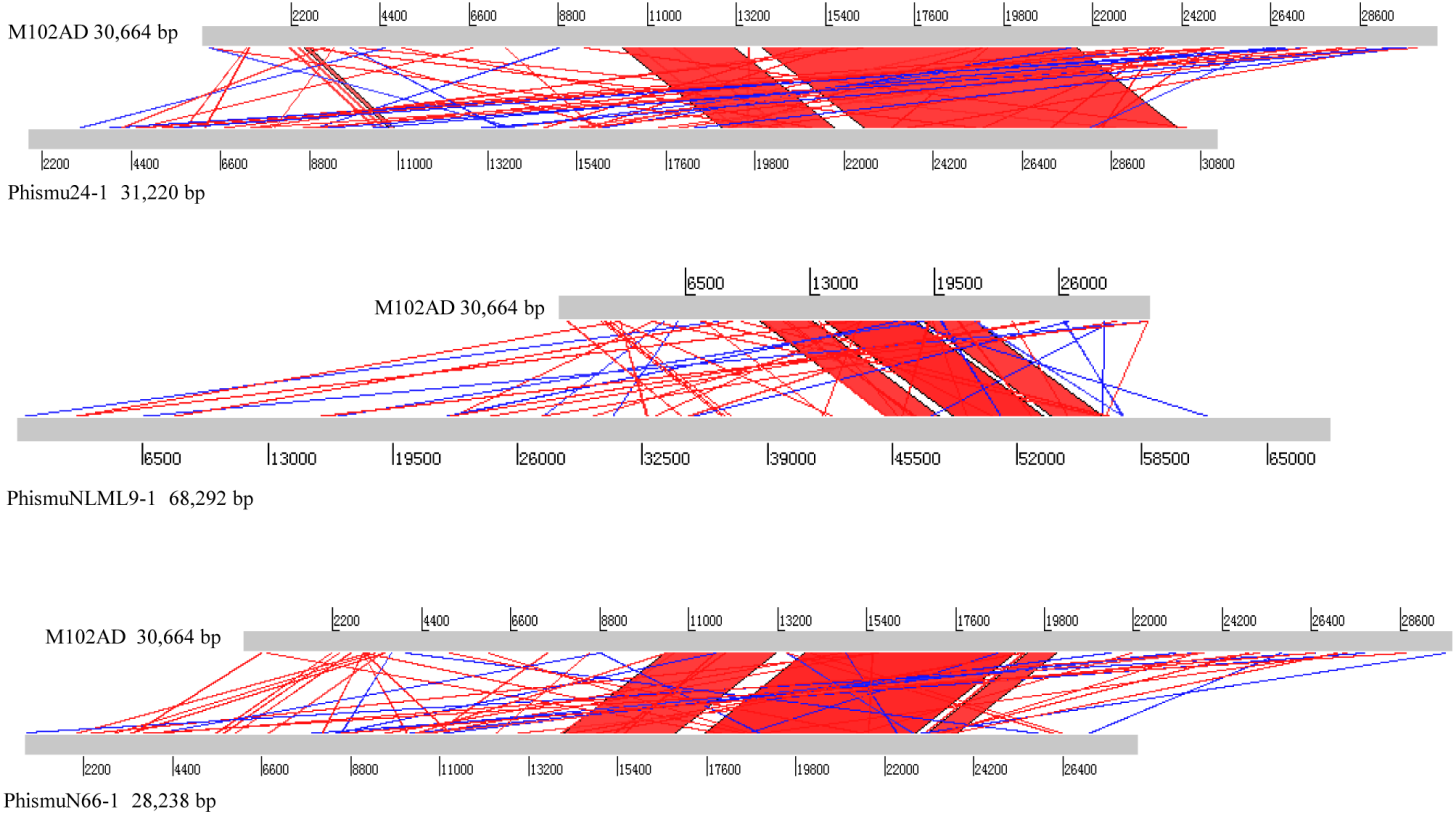
Sequence comparisons of M102AD with phismu24-1, phismuNLML9-1, and phismuN66-1. Comparisons were performed using the BLASTn and Artemis Comparison Tool visualization programs. Forward and reverse matches are colored red and blue, respectively.

The *S. mutans* phage M102AD, which has a genome length of 30,664 bp and was isolated at the University of Maryland, was chosen as a reference phage, because it has been sequenced and well annotated (Delisle et al., 2012). The prophages phismuN66-1, phismuNLML9-1, and phismu24-1 all shared sequence similarity with M102AD (Fig. 4). The linear genomic comparison showed that phismu24-1 shared two major sequence (1,199 and 466 bp) similarities of 84 and 83.2% identity, respectively, with M102AD at the nucleotide level. BLASTn comparison of phismuNLML9-1 and M102AD revealed three major sequences (666, 454, and 423 bp) with 85.6, 82.9, and 82.7% identity at the nucleotide level. PhismuN66-1 shared three major sequences (749, 473, and 124 bp) with 85.6, 83.6, and 84.2% identity in comparison with M102AD genomes.

### Summary of features of *S. mutans* prophage genomic sequences

**Figure 5:**
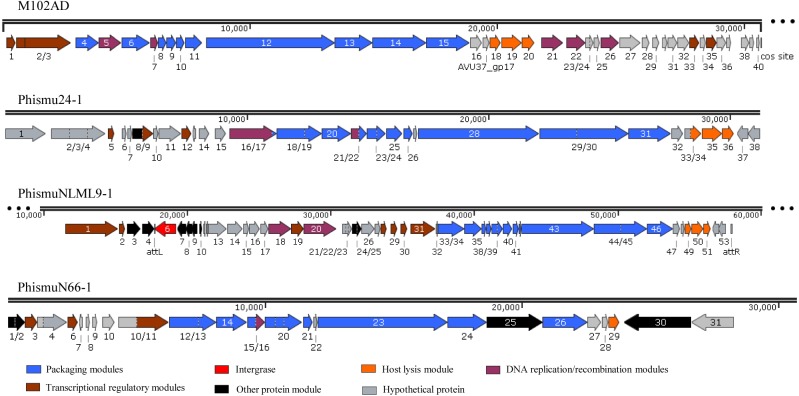
Genetic organization of the open reading frame (ORF) regions in the prophages M102AD, phismu24-1, phismuNLML9-1, and phismuN66-1. Prophage genes are grouped into seven major gene cluster modules: packaging and structural modules, integrase, host lysis module, DNA replication/recombination module, transcriptional regulatory module, other protein module, and hypothetical protein module. Corresponding genes are indicated with the same color. The line numbers indicate genomic position. The predicted ORF orientations are indicated by horizontal arrows, and arrow numbers indicate the ORF region in the genomic sequence. The figure was drawn using SnapGene (http://www.snapgene.com; GSL Biotech, Chicago, IL, USA).

Three intact prophages (phismu NLML9-1, phismu N66-1, and phismu 24-1) were identified in *S. mutans*, and all three prophages in Cluster B closely resembled the genome of M102AD. All of the ORFs of the prophages were predicted and annotated by PHASTER, GeneMarkS, and BLASP. PhismuNLML9-1, phismu24-1, and phismu66-1 exhibited the characteristic modular arrangement of the M102AD phage, including packaging and structural modules, integrase module, host lysis module, DNA replication/recombination module, transcriptional regulatory module, other protein module, and hypothetical protein module. The integrase gene was identified only in prophage phismuNLML9-1 (Fig. 5).

A total of 37 ORFs were identified in the genome of phismu24-1 (Table S2). Of the 37 ORFs, 20 were assigned a putative function. No transfer RNA (tRNA) was found in the genome of phage phismu24-1. Three significant host lysis module regions were observed in ORF 34–36. ORF 34 encodes a putative holin protein, which can form pores in cytoplasmic membranes and release toxins and other proteins or contribute to biofilm formation (Saier & Reddy, 2015). ORF 35 and ORF 36 encode putative endolysin proteins, which can digest the bacterial cell wall for phage progeny release and may have the potential to be used as antibacterial agents (Fenton et al., 2010; Fischetti, 2008). A major head and major tail protein were identified from the products of ORF 20 and ORF 25, respectively. ORF 16 and ORF 21 are predicted to be DNA replication modules, the products of which are similar to the putative large subunit of terminase and the putative DNA packaging protein.

The prophage phismuNLML9-1 contains 54 phage-related genes (Table S3). Most of the ORFs of phismuNLML9-1 are flanked by a 13-bp repeat, indicative of *attL* and *attR* sites (Fig. 5). Most temperate phages enter the lytic cycle depending on the integrase gene, which functions in chromosomal integration and excision (Smith & Thorpe, 2002). Integrase genes were identified in ORF 6 of the prophage phismuNLML9-1, suggesting that phismuNLML9-1 has the ability to enter a lysogenic replication cycle.

No putative tRNA or transfer-messenger RNA was recognized. A putative holin protein was encoded by ORF 34 and a putative endolysin by ORF 35 and ORF 36. ORF 18, ORF 9, and ORF 20 are predicted to encompass the replication module.

The phismu66-1 prophage genome contains 31 ORFs (Table S4). ORF 30 encodes the ABC transporter or permease protein, which functions as a multiple sugar metabolism transporter and is a promising target for antimicrobial strategies in *S. mutans* (Nagayama et al., 2014). ORF 25 encodes a host specificity protein and ORF 29 encodes a lysin-holin protein. In addition, the packaging and structural modules contained ORF 12, ORF 13, ORF 15, ORF 17–21, ORF 23, ORF 24, and ORF 26, and the transcriptional regulatory module was encoded by ORF 3, ORF 6, and ORF 11.

Horizontal gene transfer plays an important role in the adaptation and evolution of prokaryotes, and bacteriophages, as mobile genetic elements, enable horizontal gene transfer. In our study, we found that ORF 4 of phismuNLML9-1 and ORF 30 of phismu66-1 both encode an ABC transporter/permease protein, which is a virulence protein associated with the development of spontaneous resistance to compound 103 in *S. aureus* strains (Morisaki et al., 2016). Many unknown functional hypothetical proteins may play an important role in the acquisition of a specialized set of genes via prophages and horizontal transfer in *S. mutans*.

### Mapping of the genomic location and comparative analysis of *S. mutans* prophages

**Figure 6:**
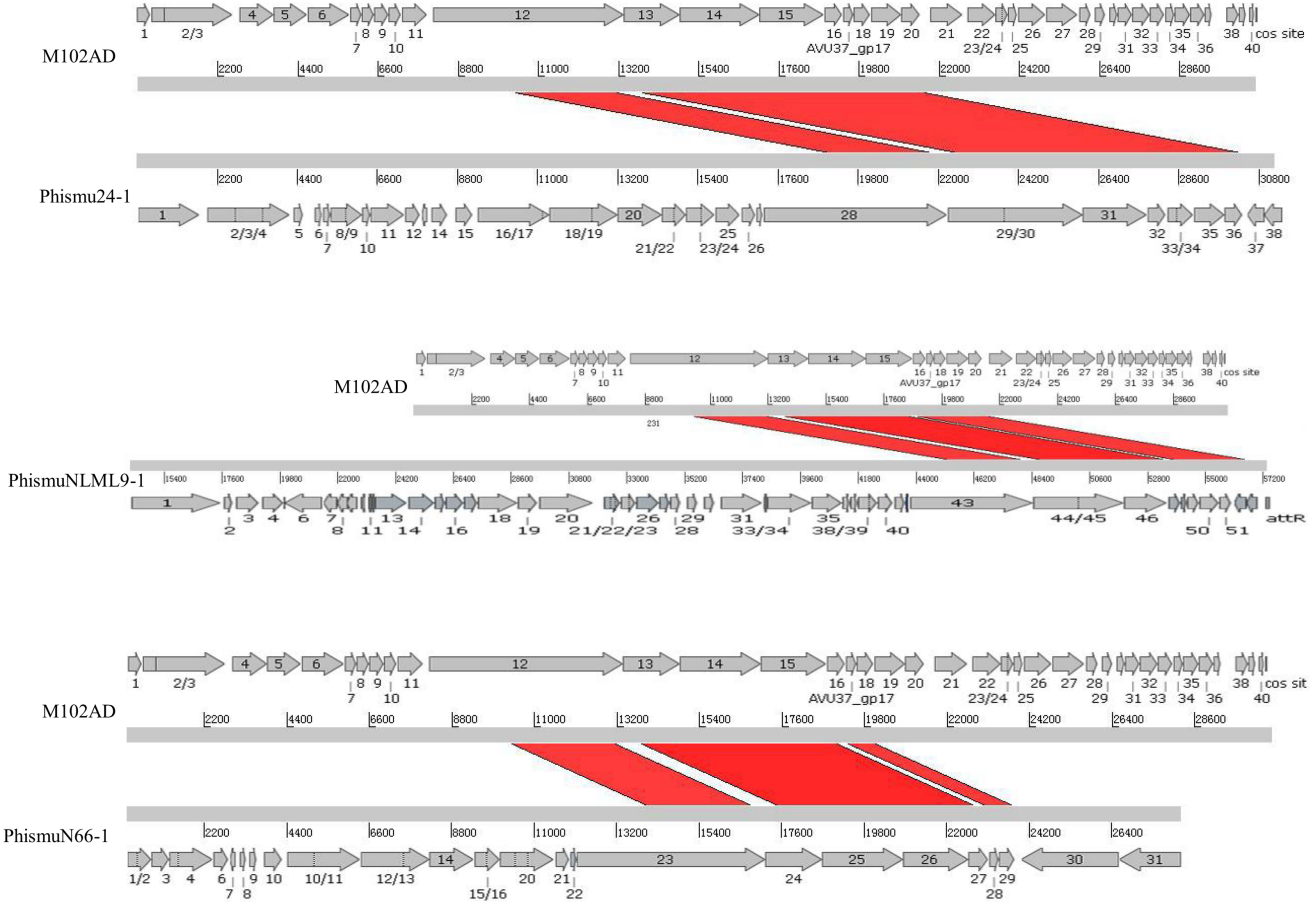
Schematic representation of the genomic organization and ORF regions of S. mutans phage M102AD compared to the prophages phismu24-1, phismuNLML9-1, and phismuN66-1. The lines represent phage/prophage genomes, and arrows represent ORFs. Regions connected by red shading represent conserved genomic identity.

We oriented and mapped the ORFs located in the genomic sequences of M102AD, phismu24-1, phismuNLML9-1, and phismuN66-1 (Fig. 6). A total of nine ORFs were located in the M102AD, phismuNLML9-1, and phismu24-1 conserved regions, and seven ORFs were located in the conserved regions of phismuN66-1. The major conserved regions of M102AD included ORFs 12–20, which encode a putative tape measure protein, putative tail protein, putative receptor-binding protein, putative minor structural protein, hypothetical protein, hypothetical protein, putative holin, putative endolysin, and putative endolysin, respectively. According to their putative functions, the ORFs were assigned to packaging/structural modules, hypothetical protein modules, and the host lysis module (Tables S5 and S6). A total of nine ORFs were identified in the conserved regions of phismu24-1, including ORF 28 and 29 (putative tail component proteins), ORF 30 (tail-host specificity protein), ORF 31 (tail protein), ORF 32 and 33 (hypothetical proteins), ORF 34 (putative holin), and ORF 35 and 36 (putative endolysins). These ORFs shared a region varying between 57.68 and 95% identity with M102AD (Table S5). The phismuNLML9-1 sequence contained nine ORFs similar to M102AD: ORF 43 and 44 (putative tail component proteins), ORF 45 (tail-host specificity protein), ORF 46 (tail protein), ORF 47 and 48 (hypothetical proteins), ORF 49 (putative holin), ORF 50 (hypothetical protein), and ORF 51 (putative endolysin). The protein sequence identity of these ORFs varied between 57.73 and 94% with M102AD (Table S5). The phismuN66-1 sequence contained seven ORFs similar to M102AD: ORF 23 and 24 (putative tail component proteins), ORF 25 (tail-host specificity protein), ORF 26 (tail protein), ORF 27 and 28 (hypothetical proteins), and ORF 29 (putative holin). The protein sequence identity of the ORFs varied between 62 and 95% with M102AD. PhismuN66-1 lost two putative endolysin ORFs compared to other prophages (Table S5).

## Conclusions

In conclusion, our genome sequencing data analyses identified 35 prophage-like elements present in the genome of *S. mutans*, all of which were identified for the first time. Genomic analysis of prophages revealed that those belonging to the same cluster displayed sequence similarities. The genomes and genetic information of phismuNLML9-1, phismu24-1, and phismu66-1 prophages were analyzed, identifying putative ORFs and functional regions. To the best of our knowledge, this is the first systematic analysis of *S. mutans* prophages.

##  Supplemental Information

10.7717/peerj.4057/supp-1 Table S1 Putative prophages detected in *Streptococcus mutans* strain using PHAST

10.7717/peerj.4057/supp-2 Table S2 Phismun24-1 genome sequence annotations

10.7717/peerj.4057/supp-3 Table S3 PhismunNLML9-1 genome sequence annotations

10.7717/peerj.4057/supp-4 Table S4 Phismun66-1 genome sequence annotations

10.7717/peerj.4057/supp-5 Table S5 The genomic identity and protein identity

10.7717/peerj.4057/supp-6 Table S6 Function modules and conserved ORFs

10.7717/peerj.4057/supp-7 Figure S1 Genome sequence comparison of representative clusterA, clusterB and clusterC** prophages Comparisons were performed using the Blastn and Artemis Comparison Tool visualisation programs. Forward and reverse matches are coloured in red and blue, respectively.

## Additional Information and Declarations

### Data Availability

The following information was supplied regarding data availability: The accession numbers can be found in Table 1.
