# Supplementary material for: Comparative analysis of prophages in Streptococcus mutans genomes
Source: PeerJ. 2017 Nov 17;5:e4057. doi: 10.7717/peerj.4057 (PMC5695247; doi:10.7717/peerj.4057)
Supplement: Table S1 [file peerj-05-4057-s001.docx]

S1 Table: Putative prophages detected in *Streptococcus mutans* strain using PHAST

| Sequence number | Organism | Strain | Accession number | No. putative  Prophages |
| --- | --- | --- | --- | --- |
| 1 | *Streptococcus mutans* UA159 | UA159 | NC_004350.2/AE014133.2 | 1 |
| 2 | *Streptococcus mutans* NN2025 | NN2025 | NC_013928.1/AP010655.1 | 0 |
| 3 | *Streptococcus mutans* GS-5 | GS-5 | NC_018089.1/CP003686.1 | 0 |
| 4 | *Streptococcus mutans* LJ23 | LJ23 | NC_017768.1/AP012336.1 | 0 |
| 5 | *Streptococcus mutans* UA159-FR | UA159-FR | NZ_CP007016.1/CP007016.1 | 1 |
| 6 | *Streptococcus mutans* NG8 | NG8 | NZ_CP013237.1/CP013237.1 | 8 |
| 7 | *Streptococcus mutans* TCI-101 | TCI-101 | AGKF00000000.1 | 0 |
| 8 | *Streptococcus mutans* TCI-109 | TCI-109 | AGKG00000000.1 | 0 |
| 9 | *Streptococcus mutans* TCI-11 | TCI-11 | AGKH00000000.1 | 0 |
| 10 | *Streptococcus mutans* TCI-110 | TCI-110 | AGKI00000000.1 |  |
| 11 | *Streptococcus mutans* TCI-116 | TCI-116 | AGKJ00000000.1 | 0 |
| 12 | *Streptococcus mutans* TCI-120 | TCI-120 | AGKK00000000.1 | 0 |
| 13 | *Streptococcus mutans* TCI-123 | TCI-123 | AGKL00000000.1 | 0 |
| 14 | *Streptococcus mutans* TCI-125 | TCI-125 | AGKM00000000.1 | 0 |
| 15 | *Streptococcus mutans* TCI-138 | TCI-138 | AGKN00000000.1 | 0 |
| 16 | *Streptococcus mutans* TCI-143 | TCI-143 | AGKO00000000.1 | 0 |
| 17 | *Streptococcus mutans* TCI-145 | TCI-145 | AGKP00000000.1 | 0 |
| 18 | *Streptococcus mutans* TCI-146 | TCI-146 | AGKE00000000.1 | 0 |
| 19 | *Streptococcus mutans* TCI-148 | TCI-148 | AGKQ00000000.1 | 0 |
| 20 | *Streptococcus mutans* TCI-149 | TCI-149 | AGKR00000000.1 | 0 |
| 21 | *Streptococcus mutans* TCI-151 | TCI-151 | AGKS00000000.1 | 0 |
| 22 | *Streptococcus mutans* TCI-152 | TCI-152 | AGKT00000000.1 | 0 |
| 23 | *Streptococcus mutans* TCI-153 | TCI-153 | AGKU00000000.1 | 0 |
| 24 | *Streptococcus mutans* TCI-154 | TCI-154 | AGKV00000000.1 | 0 |
| 25 | *Streptococcus mutans* TCI-162 | TCI-162 | AGKW00000000.1 | 0 |
| 26 | *Streptococcus mutans* TCI-163 | TCI-163 | AGKX00000000.1 | 0 |
| 27 | *Streptococcus mutans* TCI-164 | TCI-164 | AGKY00000000.1 | 0 |
| 28 | *Streptococcus mutans* TCI-169 | TCI-169 | AGKZ00000000.1 | 0 |
| 29 | *Streptococcus mutans* TCI-170 | TCI-170 | AGLA00000000.1 | 0 |
| 30 | *Streptococcus mutans* TCI-173 | TCI-173 | AGLB00000000.1 | 0 |
| 31 | *Streptococcus mutans* TCI-176 | TCI-176 | AGLC00000000.1 | 0 |
| 32 | *Streptococcus mutans* TCI-177 | TCI-177 | AGLD00000000.1 | 0 |
| 33 | *Streptococcus mutans* TCI-179 | TCI-179 | AGLE00000000.1 | 0 |
| 34 | *Streptococcus mutans* TCI-187 | TCI-187 | AGLF00000000.1 | 0 |
| 35 | *Streptococcus mutans* TCI-191 | TCI-191 | AGLG00000000.1 | 0 |
| 36 | *Streptococcus mutans* TCI-196 | TCI-196 | AGLH00000000.1 | 0 |
| 37 | *Streptococcus mutans* TCI-201 | TCI-201 | AGLI00000000.1 | 0 |
| 38 | *Streptococcus mutans* TCI-202 | TCI-202 | AGLJ00000000.1 | 0 |
| 39 | *Streptococcus mutans* TCI-204 | TCI-204 | AGLK00000000.1 | 0 |
| 40 | *Streptococcus mutans* TCI-210 | TCI-210 | AGLL00000000.1 | 0 |
| 41 | *Streptococcus mutans* TCI-212 | TCI-212 | AGLM00000000.1 | 0 |
| 42 | *Streptococcus mutans* TCI-218 | TCI-218 | AGLN00000000.1 | 0 |
| 43 | *Streptococcus mutans* TCI-219 | TCI-219 | AGLO00000000.1 | 0 |
| 44 | *Streptococcus mutans* TCI-220 | TCI-220 | AGLP00000000.1 | 0 |
| 45 | *Streptococcus mutans* TCI-222 | TCI-222 | AGLQ00000000.1 | 0 |
| 46 | *Streptococcus mutans* TCI-223 | TCI-223 | AGLR00000000.1 | 0 |
| 47 | *Streptococcus mutans* TCI-224 | TCI-224 | AGLS00000000.1 | 0 |
| 48 | *Streptococcus mutans* TCI-227 | TCI-227 | AGLT00000000.1 | 0 |
| 49 | *Streptococcus mutans* TCI-228 | TCI-228 | AGLU00000000.1 | 0 |
| 50 | *Streptococcus mutans* TCI-234 | TCI-234 | AGLV00000000.1 | 0 |
| 51 | *Streptococcus mutans* TCI-239 | TCI-239 | AGLW00000000.1 | 0 |
| 52 | *Streptococcus mutans* TCI-242 | TCI-242 | AGLX00000000.1 | 0 |
| 53 | *Streptococcus mutans* TCI-243 | TCI-243 | AGLY00000000.1 | 0 |
| 54 | *Streptococcus mutans* TCI-244 | TCI-244 | AGLZ00000000.1 | 0 |
| 55 | *Streptococcus mutans* TCI-249 | TCI-249 | AGMA00000000.1 | 0 |
| 56 | *Streptococcus mutans* TCI-256 | TCI-256 | AGMB00000000.1 | 0 |
| 57 | *Streptococcus mutans* TCI-260 | TCI-260 | AGMC00000000.1 | 0 |
| 58 | *Streptococcus mutans* TCI-264 | TCI-264 | AGMD00000000.1 | 0 |
| 59 | *Streptococcus mutans* TCI-267 | TCI-267 | AGME00000000.1 | 0 |
| 60 | *Streptococcus mutans* TCI-268 | TCI-268 | AGMF00000000.1 | 0 |
| 61 | *Streptococcus mutans* TCI-278 | TCI-278 | AGMG00000000.1 | 0 |
| 62 | *Streptococcus mutans* TCI-279 | TCI-279 | AGMH00000000.1 | 0 |
| 63 | *Streptococcus mutans* TCI-280 | TCI-280 | AGMI00000000.1 | 0 |
| 64 | *Streptococcus mutans* TCI-289 | TCI-289 | AGMJ00000000.1 | 0 |
| 65 | *Streptococcus mutans* TCI-292 | TCI-292 | AGMK00000000.1 | 0 |
| 66 | *Streptococcus mutans* TCI-294 | TCI-294 | AGML00000000.1 | 0 |
| 67 | *Streptococcus mutans* TCI-298 | TCI-298 | AGMM00000000.1 | 0 |
| 68 | *Streptococcus mutans* TCI-30 | TCI-30 | AGMN00000000.1 | 0 |
| 69 | *Streptococcus mutans* TCI-399 | TCI-399 | AGMO00000000.1 | 0 |
| 70 | *Streptococcus mutans* TCI-400 | TCI-400 | AGMP00000000.1 | 0 |
| 71 | *Streptococcus mutans* TCI-51 | TCI-51 | AGMQ00000000.1 | 0 |
| 72 | *Streptococcus mutans* TCI-62 | TCI-62 | AGMR00000000.1 | 0 |
| 73 | *Streptococcus mutans* TCI-70 | TCI-70 | AGMS00000000.1 | 0 |
| 74 | *Streptococcus mutans* TCI-75 | TCI-75 | AGMT00000000.1 | 0 |
| 75 | *Streptococcus mutans* TCI-78 | TCI-78 | AGMU00000000.1 | 0 |
| 76 | *Streptococcus mutans* TCI-82 | TCI-82 | AGMV00000000.1 | 0 |
| 77 | *Streptococcus mutans* TCI-85 | TCI-85 | AGMW00000000.1 | 0 |
| 78 | *Streptococcus mutans* TCI-86 | TCI-86 | AGMX00000000.1 | 0 |
| 79 | *Streptococcus mutans* TCI-92 | TCI-92 | AGMY00000000.1 | 0 |
| 80 | *Streptococcus mutans* TCI-96 | TCI-96 | AGMZ00000000.1 | 0 |
| 81 | *Streptococcus mutans* TCI-99 | TCI-99 | AGNA00000000.1 | 0 |
| 82 | *Streptococcus mutans* S1B | S1B | AHRC00000000.1 | 0 |
| 83 | *Streptococcus mutans* SA41 | SA41 | AHRE00000000.1 | 0 |
| 84 | *Streptococcus mutans* SF12 | SF12 | AHRF00000000.1 | 0 |
| 85 | *Streptococcus mutans* R221 | R221 | AHRG00000000.1 | 2 |
| 86 | *Streptococcus mutans* M230 | M230 | AHRH00000000.1 | 2 |
| 87 | *Streptococcus mutans* 15JP3 | 15JP3 | AHRJ00000000.1 | 0 |
| 88 | *Streptococcus mutans* 4SM1 | 4SM1 | AHRL00000000.1 | 0 |
| 89 | *Streptococcus mutans* 2ST1 | 2ST1 | AHRN00000000.1 | 0 |
| 90 | *Streptococcus mutans* 4VF1 | 4VF1 | AHRQ00000000.1 | 0 |
| 91 | *Streptococcus mutans* 15VF2 | 15VF2 | AHRR00000000.1 | 0 |
| 92 | *Streptococcus mutans* 11VS1 | 11VS1 | AHRT00000000.1 | 0 |
| 93 | *Streptococcus mutans* 5SM3 | 5SM3 | AHRU00000000.1 | 0 |
| 94 | *Streptococcus mutans* NFSM2 | NFSM2 | AHRV00000000.1 | 0 |
| 95 | *Streptococcus mutans* A9 | A9 | AHRX00000000.1 | 0 |
| 96 | *Streptococcus mutans* N29 | N29 | AHRY00000000.1 | 2 |
| 97 | *Streptococcus mutans* NMT4863 | NMT4863 | AHRZ00000000.1 | 0 |
| 98 | *Streptococcus mutans* T4 | T4 | AHSE00000000.1 | 0 |
| 99 | *Streptococcus mutans* NFSM1 | NFSM1 | AHSG00000000.1 | 1 |
| 100 | *Streptococcus mutans* NV1996 | NV1996 | AHSN00000000.1 | 0 |
| 101 | *Streptococcus mutans* SF14 | SF14 | AHSQ00000000.1 | 1 |
| 102 | *Streptococcus mutans* SM6 | SM6 | AHSR00000000.1 | 0 |
| 103 | *Streptococcus mutans* U2A | U2A | AHSU00000000.1 | 1 |
| 104 | *Streptococcus mutans* NLML8 | NLML8 | AHSV00000000.1 | 0 |
| 105 | *Streptococcus mutans* 14D | 14D | AHSY00000000.1 | 0 |
| 106 | *Streptococcus mutans* 21 | 21 | AHSZ00000000.1 | 1 |
| 107 | *Streptococcus mutans* B | B | AHTB00000000.1 | 1 |
| 108 | *Streptococcus mutans* SM1 | SM1 | AHTD00000000.1 | 1 |
| 109 | *Streptococcus mutans* 8ID3 | 8ID3 | AHRB00000000.1 | 0 |
| 110 | *Streptococcus mutans* SA38 | SA38 | AHRD00000000.1 | 0 |
| 111 | *Streptococcus mutans* OMZ175 | OMZ175 | AHRI00000000.1 | 0 |
| 112 | *Streptococcus mutans* 1SM1 | 1SM1 | AHRK00000000.1 | 0 |
| 113 | *Streptococcus mutans* 3SN1 | 3SN1 | AHRM00000000.1 | 1 |
| 114 | *Streptococcus mutans* 11A1 | 11A1 | AHRO00000000.1 | 0 |
| 115 | *Streptococcus mutans* 11SSST2 | 11SSST2 | AHRP00000000.1 | 1 |
| 116 | *Streptococcus mutans* 2VS1 | 2VS1 | AHRS00000000.1 | 0 |
| 117 | *Streptococcus mutans* NVAB | NVAB | AHRW00000000.1 | 0 |
| 118 | *Streptococcus mutans* A19 | A19 | AHSA00000000.1 | 0 |
| 119 | *Streptococcus mutans* U138 | U138 | AHSB00000000.1 | 0 |
| 120 | *Streptococcus mutans* G123 | G123 | AHSC00000000.1 | 0 |
| 121 | *Streptococcus mutans* M21 | M21 | AHSD00000000.1 | 0 |
| 122 | *Streptococcus mutans* N34 | N34 | AHSF00000000.1 | 0 |
| 123 | *Streptococcus mutans* NLML4 | NLML4 | AHSH00000000.1 | 0 |
| 124 | *Streptococcus mutans* NLML5 | NLML5 | AHSI00000000.1 | 0 |
| 125 | *Streptococcus mutans* NLML9 | NLML9 | AHSJ00000000.1 | 2 |
| 126 | *Streptococcus mutans* M2A | M2A | AHSK00000000.1 | 0 |
| 127 | *Streptococcus mutans* N3209 | N3209 | AHSL00000000.1 | 0 |
| 128 | *Streptococcus mutans* N66 | N66 | AHSM00000000.1 | 2 |
| 129 | *Streptococcus mutans* W6 | W6 | AHSO00000000.1 | 1 |
| 130 | *Streptococcus mutans* SF1 | SF1 | AHSP00000000.1 | 1 |
| 131 | *Streptococcus mutans* ST1 | ST1 | AHSS00000000.1 | 0 |
| 132 | *Streptococcus mutans* ST6 | ST6 | AHST00000000.1 | 1 |
| 133 | *Streptococcus mutans* NLML1 | NLML1 | AHSW00000000.1 | 1 |
| 134 | *Streptococcus mutans* 1ID3 | 1ID3 | AHSX00000000.1 | 0 |
| 135 | *Streptococcus mutans* 66-2A | 66-2A | AHTA00000000.1 | 0 |
| 136 | *Streptococcus mutans* SM4 | SM4 | AHTC00000000.1 | 0 |
| 137 | *Streptococcus mutans* 24 | 24 | AHTE00000000.1 | 2 |
| 138 | *Streptococcus mutans* U2B | U2B | AGWE00000000.1 | 0 |
| 139 | *Streptococcus mutans* 5DC8 | 5DC8 | AOBX00000000.1 | 1 |
| 140 | *Streptococcus mutans* KK21 | KK21 | AOBY00000000.1 | 1 |
| 141 | *Streptococcus mutans* AC4446 | AC4446 | AOCA00000000.1 | 0 |
| 142 | *Streptococcus mutans* ATCC 25175 | ATCC 25175 | AOCB00000000.1 | 0 |
| 143 | *Streptococcus mutans* NCTC 11060 | NCTC 11060 | AOCC00000000.1 | 0 |
| 144 | *Streptococcus mutans* DSM 20523 | DSM 20523 | AQWT00000000.1 | 0 |
| 145 | *Streptococcus mutans* PKUSS-HG01 | PKUSS-HG01 | AXSW00000000.1 | 0 |
| 146 | *Streptococcus mutans* PKUSS-LG01 | PKUSS-LG01 | AXSX00000000.1 | 0 |
| 147 | *Streptococcus mutans* B05Sm11 | B05Sm11 | ALYO00000000.1 | 0 |
| 148 | *Streptococcus mutans* B13Sm1 | B13Sm1 | ALYP00000000.1 | 0 |
| 149 | *Streptococcus mutans* B12Sm1 | B12Sm1 | ALYQ00000000.1 | 0 |
| 150 | *Streptococcus mutans* B084SM-A | B084SM-A | ALYR00000000.1 | 0 |
| 151 | *Streptococcus mutans* B107SM-B | B107SM-B | ALYS00000000.1 | 0 |
| 152 | *Streptococcus mutans* B07Sm2 | B07Sm2 | ALYT00000000.1 | 0 |
| 153 | *Streptococcus mutans* B09Sm1 | B09Sm1 | ALYU00000000.1 | 0 |
| 154 | *Streptococcus mutans* B24Sm2 | B24Sm2 | ALYV00000000.1 | 0 |
| 155 | *Streptococcus mutans* B102SM-B | B102SM-B | ALYW00000000.1 | 0 |
| 156 | *Streptococcus mutans* B112SM-A | B112SM-A | ALYX00000000.1 | 0 |
| 157 | *Streptococcus mutans* B04Sm5 | B04Sm5 | ALYY00000000.1 | 0 |
| 158 | *Streptococcus mutans* B082SM-A | B082SM-A | ALYZ00000000.1 | 0 |
| 159 | *Streptococcus mutans* B06Sm2 | B06Sm2 | ALZA00000000.1 | 0 |
| 160 | *Streptococcus mutans* B85SM-B | B85SM-B | ALZB00000000.1 | 0 |
| 161 | *Streptococcus mutans* B88SM-A | B88SM-A | ALZC00000000.1 | 0 |
| 162 | *Streptococcus mutans* str. B16 P Sm1 | B16 P Sm1 | ALZD00000000.1 | 0 |
| 163 | *Streptococcus mutans* B23Sm1 | B23Sm1 | ALZE00000000.1 | 0 |
| 164 | *Streptococcus mutans* B111SM-A | B111SM-A | ALZF00000000.1 | 0 |
| 165 | *Streptococcus mutans* B114SM-A | B114SM-A | ALZG00000000.1 | 0 |
| 166 | *Streptococcus mutans* B115SM-A | B115SM-A | ALZH00000000.1 | 0 |
| 167 | *Streptococcus mutans* | 1006_SMUT | JWGO00000000.1 | 0 |
| 168 | *Streptococcus mutans* | 1002_SMUT | JWGS00000000.1 | 0 |
| 169 | *Streptococcus mutans* | ATCC 55676 | LTAK00000000.1 | 0 |
| 170 | *Streptococcus mutans* KK23 | KK23 | AOBZ00000000.1 | 0 |
| 171 | *Streptococcus mutans* | 503_SMUT | JVEO00000000.1 | 0 |
