# Supplementary material for: Comparative analysis of prophages in Streptococcus mutans genomes
Source: PeerJ. 2017 Nov 17;5:e4057. doi: 10.7717/peerj.4057 (PMC5695247; doi:10.7717/peerj.4057)
Supplement: Table S2 [file peerj-05-4057-s002.docx]

Table S2: Phismun24-1 genome sequence annotations.

| **ORF** | **ORF _POSITION** | **BLAST_HIT** | **E-VALUE** |
| --- | --- | --- | --- |
| ORF 1 | 1..1659 | hypothetical protein | 5e-05 |
| ORF 2 | 1884..2723 | DegV domain-containing protein | 9e-15 |
| ORF 3 | 2716..3573 | hypothetical protein | 1e-160 |
| ORF 4 | 3548..4147 | hypothetical protein | 1e-108 |
| ORF 5 | 4244..4519 | DNA-binding HU protein | 8e-29 |
| ORF 6 | 4833..5030 | hypothetical | 0.0 |
| ORF 7 | 5005..5250 | hypothetical protein | 1e-30 |
| ORF 8 | 5247..5678 | rus | 5e-35 |
| ORF 9 | 5671..6135 | Cytosine specific DNA methyltransferase | 6e-77 |
| ORF 10 | 6108..6359 | hypothetical protein | 2e-06 |
| ORF 11 | 6356..7282 | hypothetical protein | 7e-10 |
| ORF 12 | 7303..7716 | putative transcriptional activator | 9e-28 |
| ORF 13 | Complement  (7760..7891) | hypothetical | 0.0 |
| ORF 14 | 8029..8460 | hypothetical protein | 2e-32 |
| ORF 15 | 8677..9138 | hypothetical protein | 4e-66 |
| ORF 16 | 9284..11059 | putative large subunit of the terminase | 0.0 |
| ORF 17 | 11040..11237 | putative head-tail joining protein | 2e-12 |
| ORF 18 | 11255..12409 | putative portal protein | 6e-158 |
| ORF 19 | 12396..13097 | putative scaffolding protein | 2e-82 |
| ORF 20 | 13097..14302 | major head protein | 1e-132 |
| ORF 21 | 14322..14648 | putative DNA packaging protein | 7e-24 |
| ORF 22 | 14641..14988 | putative head-tail joining protein | 5e-22 |
| ORF 23 | 14990..15409 | putative tail component protein | 2e-35 |
| ORF 24 | 15393..15764 | putative tail component protein | 2e-22 |
| ORF 25 | 15771..16436 | major tail protein | 2e-45 |
| ORF 26 | 16496..16879 | putative tail component protein | 2e-06 |
| ORF 27 | 16903..17067 | hypothetical protein | 4e-08 |
| ORF 28 | 17079..22112 | putative tail component protein | 0.0 |
| ORF 29 | 22128..23657 | putative tail component protein | 6e-61 |
| ORF 30 | 23654..25810 | tail-host specificity protein | 5e-59 |
| ORF 31 | 25811..27568 | tail protein | 5e-54 |
| ORF 32 | 27590..28093 | hypothetical protein | 3e-61 |
| ORF 33 | 28111..28383 | hypothetical protein | 1e-39 |
| ORF 34 | 28380..28847 | putative holin | 1e-72 |
| ORF 35 | 28861..29682 | putative endolysin | 2e-147 |
| ORF 36 | 29682..30197 | putative endolysin | 2e-86 |
| ORF 37 | Complement  (30340..30771) | hypothetical protein | 2e-38 |
| ORF 38 | Complement  (30800..31222) | hypothetical protein | 3e-59 |

ORF, open reading frame.
