# Supplementary material for: Comparative analysis of prophages in Streptococcus mutans genomes
Source: PeerJ. 2017 Nov 17;5:e4057. doi: 10.7717/peerj.4057 (PMC5695247; doi:10.7717/peerj.4057)
Supplement: Table S3 [file peerj-05-4057-s003.docx]

Table S3: PhismunNLML9-1 genome sequence annotations.

| **ORF** | **ORF _POSITION** | **BLAST_HIT** | **E-VALUE** |
| --- | --- | --- | --- |
| ORF 1 | 11536..15165 | DNA-directed RNA polymerase II subunit | 2e-59 |
| ORF 2 | 15292..15675 | DNA binding protein | 1e-76 |
| ORF 3 | 15808..16749 | ABC transporter ATP-binding protein | 1e-175 |
| ORF 4 | 16883..17716 | ABC transporter | 3e-153 |
| ORF 5 | 17746..17764 | attL    GCTTTTACGCTTATAGAAA | 0.0 |
| ORF 6 | complement(17784..19226) | phage integrase protein | 0.0 |
| ORF 7 | complement(19358..19879) | phage protein | 4e-32 |
| ORF 8 | complement(19936..20316) | gp3 | 7e-30 |
| ORF 9 | complement(20309..20671) | peptidase S24-like protein | 1e-22 |
| ORF 10 | 20854..21060 | helix-turn-helix protein | 2e-19 |
| ORF 11 | 21162..21302 | hypothetical protein | 1e-05 |
| ORF 12 | 21316..21453 | phage protein | 3e-11 |
| ORF 13 | 21465..22781 | hypothetical protein | 9e-167 |
| ORF 14 | 22801..23883 | hypothetical protein | 5e-108 |
| ORF 15 | 23925..24350 | hypothetical protein | 4e-40 |
| ORF 16 | 24353..25084 | hypothetical protein | 1e-73 |
| ORF 17 | 25106..25681 | hypothetical protein | 8e-31 |
| ORF 18 | 25681..27264 | DEAD box family helicase | 0.0 |
| ORF 19 | 27268..28137 | putative DNA methylase | 6e-69 |
| ORF 20 | 28147..30402 | DNA primase | 0.0 |
| ORF 21 | 30827..31069 | hypothetical | 0.0 |
| ORF 22 | 31050..31274 | hypothetical | 0.0 |
| ORF 23 | 31249..31494 | hypothetical protein | 8e-31 |
| ORF 24 | 31515..31925 | rus | 1e-34 |
| ORF 25 | 31922..32143 | hypothetical protein | 2e-06 |
| ORF 26 | 32143..33072 | hypothetical protein | 5e-12 |
| ORF 27 | 33075..33503 | hypothetical | 0.0 |
| ORF 28 | 33516..33929 | putative transcriptional activator | 1e-27 |
| ORF 29 | 34185..34676 | HNH endonuclease | 5e-39 |
| ORF 30 | 34900..35361 | terminase small subunit | 5e-67 |
| ORF 31 | 35573..37282 | putative large subunit of the terminase | 0.0 |
| ORF 32 | 37323..37460 | putative head-tail joining protein | 6e-07 |
| ORF 33 | 37478..38632 | putative portal protein | 6e-159 |
| ORF 34 | 38619..39320 | putative scaffolding protein | 4e-82 |
| ORF 35 | 39320..40531 | major head protein | 9e-136 |
| ORF 36 | 40551..40877 | putative DNA packaging protein | 7e-24 |
| ORF 37 | 40870..41217 | putative head-tail joining protein | 7e-22 |
| ORF 38 | 41219..41638 | putative tail component protein | 2e-35 |
| ORF 39 | 41622..41993 | putative tail component protein | 2e-22 |
| ORF 40 | 41999..42664 | major tail protein | 4e-45 |
| ORF 41 | 42724..43107 | putative tail component protein | 2e-06 |
| ORF 42 | 43131..43295 | hypothetical protein | 4e-08 |
| ORF 43 | 43307..48340 | putative tail component protein | 0.0 |
| ORF 44 | 48356..49885 | putative tail component protein | 6e-61 |
| ORF 45 | 49882..52041 | tail-host specificity protein | 5e-61 |
| ORF 46 | 52042..53847 | putative minor structural protein | 0.0 |
| ORF 47 | 53871..54374 | hypothetical protein | 4e-83 |
| ORF 48 | 54416..54664 | hypothetical protein | 4e-36 |
| ORF 49 | 54661..55128 | putative holin | 4e-71 |
| ORF 50 | 55144..55965 | putative endolysin | 1e-149 |
| ORF 51 | 55965..56480 | putative endolysin | 1e-88 |
| ORF 52 | complement(56623..57054) | hypothetical protein | 2e-38 |
| ORF 53 | complement(57070..57492) | hypothetical protein | 2e-61 |
| ORF 54 | 57876..57894 | attR    GCTTTTACGCTTATAGAAA | 0.0 |

ORF, open reading frame.
