# Supplementary material for: Comparative analysis of prophages in Streptococcus mutans genomes
Source: PeerJ. 2017 Nov 17;5:e4057. doi: 10.7717/peerj.4057 (PMC5695247; doi:10.7717/peerj.4057)
Supplement: Table S4 [file peerj-05-4057-s004.docx]

Table S4: Phismun66-1 genome sequence annotations.

| **ORF** | **ORF _POSITION** | **BLAST_HIT** | **E-VALUE** |
| --- | --- | --- | --- |
| ORF 1 | 1..246 | hypothetical protein | 8e-31 |
| ORF 2 | 243..680 | Rus, putative phage associated protein | 1e-34 |
| ORF 3 | 673..1137 | Cytosine specific DNA methyltransferase; | 2e-77 |
| ORF 4 | 1140..1361 | hypothetical protein | 2e-06 |
| ORF 5 | 1358..2284 | hypothetical protein | 8e-12 |
| ORF 6 | 2305..2718 | putative transcriptional activator | 3e-27 |
| ORF 7 | Complement  (2762..2893) | hypothetical | 0.0 |
| ORF 8 | 3038..3184 | hypothetical | 0.0 |
| ORF 9 | 3265..3462 | hypothetical protein phiadhp38 | 2e-14 |
| ORF 10 | 3678..4139 | hypothetical protein | 1e-67 |
| ORF 11 | 4285..6060 | terminase large subunit | 0.0 |
| ORF 12 | 6047..6238 | head-tail joining protein | 5e-12 |
| ORF 13 | 6256..7410 | portal protein | 3e-156 |
| ORF 14 | 7397..8098 | scaffolding protein | 5e-84 |
| ORF 15 | 8098..9303 | major capsid protein | 1e-131 |
| ORF 16 | 9323..9649 | DNA packaging protein | 7e-24 |
| ORF 17 | 9642..9989 | capsid-tail joining protein | 4e-22 |
| ORF 18 | 9991..10401 | tail component protein | 9e-28 |
| ORF 19 | 10385..10756 | tail component protein | 3e-22 |
| ORF 20 | 10720..11427 | major tail protein | 2e-44 |
| ORF 21 | 11487..11870 | tail component protein | 1e-05 |
| ORF 22 | 11894..12058 | hypothetical protein | 4e-08 |
| ORF 23 | 12070..17103 | putative tail component protein | 0.0 |
| ORF 24 | 17119..18648 | putative tail protein | 0.0 |
| ORF 25 | 18645..20801 | host specificity protein | 7e-61 |
| ORF 26 | 20802..22559 | tail protein | 3e-57 |
| ORF 27 | 22562..23083 | hypothetical protein | 1e-60 |
| ORF 28 | 23125..23373 | hypothetical protein | 3e-36 |
| ORF 29 | 23370..23813 | putative holin | 3e-62 |
| ORF 30 | Complement  (24006..26579) | ABC transporter, permease protein | 0.0 |
| ORF 31 | Complement  (26619..28238) | hypothetical protein | 3e-20 |

ORF, open reading frame.
