# Supplementary material for: Comparative analysis of prophages in Streptococcus mutans genomes
Source: PeerJ. 2017 Nov 17;5:e4057. doi: 10.7717/peerj.4057 (PMC5695247; doi:10.7717/peerj.4057)
Supplement: Table S5 [file peerj-05-4057-s005.docx]

| Table S5. The genomic identity and protein identity. | | | | | | |
| --- | --- | --- | --- | --- | --- | --- |
|  | M102AD | | Phismun24-1 | |  | |
|  | Genomic positions | ORF | Genomic positions | ORF | Genomic identity | Protein identity |
| M102AD/ Phismun24-1 | 10373-13160  13843-21600 | ORF12: putative tape measure protein  ORF13:putative tail protein  ORF14:putative receptor-binding protein  ORF15:putative minor structural protein  ORF16:hypothetical protein  ORF17:hypothetical protein  ORF18:putative holin  ORF19:putative endolysin  ORF20:putative endolysin | 19017-21804  22529-30283 | ORF28: putative tail component protein  ORF29:putative tail component protein  ORF30:tail-host specificity protein  ORF31:tail protein  ORF32:hypothetical protein  ORF33:hypothetical protein  ORF34:putative holin  ORF35:putative endolysin  ORF36:putative endolysin | 83.28%  84.06% | 57.68%  81%  95%  91%  67%  91%  90%  91%  89% |
|  | M102AD | | PhismunNLML9-1 | |  |  |
|  | Genomic positions | ORF | Genomic positions | ORF |  |  |
| M102AD/ PhismunNLML9-1 | 10394-13160  13848-18556  18892-21587 | ORF12:putative tape measure protein  ORF13:putative tail protein  ORF14:putative receptor-binding protein  ORF15:putative minor structural protein  ORF16:hypothetical protein  ORF17:hypothetical protein  ORF18:putative holin  ORF19:putative endolysin  ORF20:putative endolysin | 45266-48032  48757-53468  53855-56552 | ORF43:putative tail component protein  ORF44:putative tail component protein  ORF45:tail-host specificity protein  ORF46: tail protein  ORF47:hypothetical protein  ORF48:hypothetical protein  ORF49:putative holin  ORF50: hypothetical protein  ORF51:putative endolysin | 83.81%  85.71%  84.32% | 57.73%  82%  96%  78%  86%  90%  90%  94%  91% |
|  | M102AD | | PhismunN66-1 | |  |  |
|  | Genomic positions | ORF | Genomic positions | ORF |  |  |
| M102AD/ PhismunN66-1 | 10394-13160  13848-19065  19373-20097 | ORF12:putative tape measure protein  ORF13:putative tail protein  ORF14:putative receptor-binding protein  ORF15:putative minor structural protein  ORF16:hypothetical protein  ORF17:hypothetical protein  ORF18:putative holin | 14029-16795  17520-22737  23045-23770 | ORF23:putative tail component protein  ORF24:putative tail protein  ORF25:host specificity protein  ORF26:tail protein  ORF27:hypothetical protein  ORF28:hypothetical protein  ORF29:putative holin | 82.91%  85.64%  82.78% | 62.05%  81%  95%  90%  68%  91%  85% |
|  |  |  |  |  |  |  |
