# Supplementary material for: Comparative analysis of prophages in Streptococcus mutans genomes
Source: PeerJ. 2017 Nov 17;5:e4057. doi: 10.7717/peerj.4057 (PMC5695247; doi:10.7717/peerj.4057)
Supplement: Table S6 [file peerj-05-4057-s006.docx]

Table S6. Function modules and conserved ORFs.

| Prophages | ORFs | Function modules |
| --- | --- | --- |
| M102AD | ORF12: putative tape measure protein  ORF13:putative tail protein  ORF14:putative receptor-binding protein  ORF15:putative minor structural protein  ORF16:hypothetical protein  ORF17:hypothetical protein  ORF18:putative holin  ORF19:putative endolysin  ORF20:putative endolysin | packaging and structural modules  hypothetical protein modules  host lysis modules |
| Phismun24-1 | ORF28: putative tail component protein  ORF29:putative tail component protein  ORF30:tail-host specificity protein  ORF31:tail protein  ORF32:hypothetical protein  ORF33:hypothetical protein  ORF34:putative holin  ORF35:putative endolysin  ORF36:putative endolysin | packaging and structural modules  hypothetical protein modules  host lysis modules |
| PhismunNLML9-1 | ORF43:putative tail component protein  ORF44:putative tail component protein  ORF45:tail-host specificity protein  ORF46: tail protein  ORF47:hypothetical protein  ORF48:hypothetical protein  ORF50: hypothetical protein  ORF49:putative holin  ORF51:putative endolysin | packaging and structural modules  hypothetical protein modules  host lysis modules |
| PhismunN66-1 | ORF23:putative tail component protein  ORF24:putative tail protein  ORF25:host specificity protein  ORF26:tail protein  ORF27:hypothetical protein  ORF28:hypothetical protein  ORF29:putative holin | packaging and structural modules  hypothetical protein modules  host lysis modules |
