# Supplementary figures and images for: Comparative analysis of prophages in Streptococcus mutans genomes
Source: PeerJ. 2017 Nov 17;5:e4057. doi: 10.7717/peerj.4057 (PMC5695247; doi:10.7717/peerj.4057)

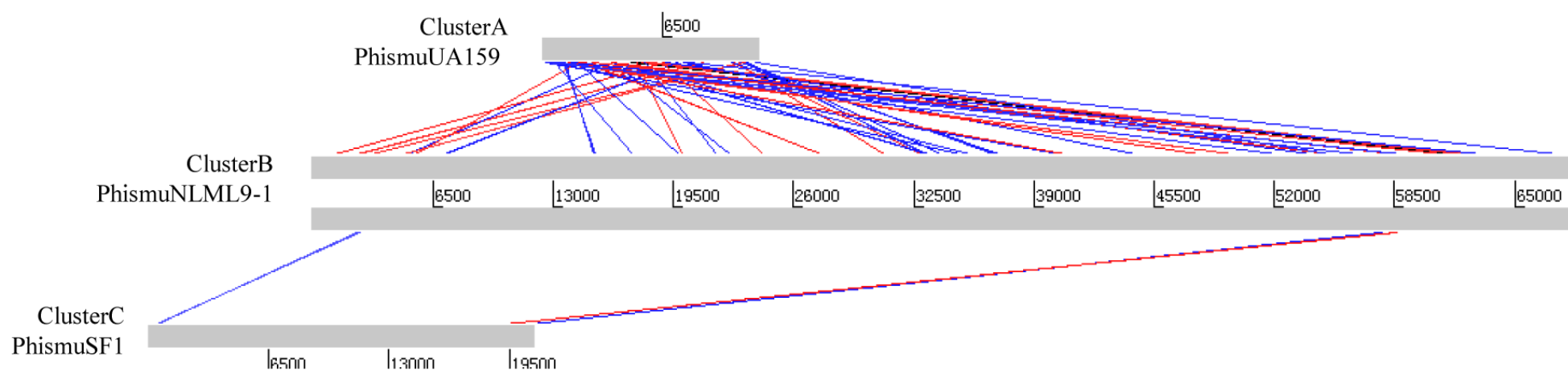

Supplement: Figure S1 — Comparisons were performed using the Blastn and Artemis Comparison Tool visualisation programs. Forward and reverse matches are coloured in red and blue, respectively. [file peerj-05-4057-s007.pdf]
